# Development and implementation of a pediatric adverse childhood experiences (ACEs) and other determinants of health questionnaire in the pediatric medical home: A pilot study

**DOI:** 10.1371/journal.pone.0208088

**Published:** 2018-12-12

**Authors:** Kadiatou Koita, Dayna Long, Danielle Hessler, Mindy Benson, Karen Daley, Monica Bucci, Neeta Thakur, Nadine Burke Harris

**Affiliations:** 1 The Center for Youth Wellness, San Francisco, California, United States of America; 2 Benioff Children’s Hospital Oakland, Oakland (BCHO), University of California San Francisco, Oakland, California, United States of America; 3 Department of Family Community Medicine, University of California San Francisco (UCSF), San Francisco, California, United States of America; 4 Department of Medicine, Division of Pulmonary and Critical Care Medicine, University of California San Francisco (UCSF) School of Medicine, San Francisco, California, United States of America; Chinese Academy of Medical Sciences and Peking Union Medical College, CHINA

## Abstract

Adverse Childhood Experiences (ACEs) are associated with poor health outcomes, underlining the significance of early identification and intervention. Currently, there is no validated tool to screen for ACEs exposure in childhood. To fill this gap, we designed and implemented a pediatric ACEs questionnaire in an urban pediatric Primary Care Clinic. Questionnaire items were selected and modified based on literature review of existing childhood adversity tools. Children twelve years and under were screened via caregiver report, using the developed instrument. Cognitive interviews were conducted with caregivers, health providers, and clinic staff to assess item interpretation, clarity, and English/Spanish language equivalency. Using a rapid cycle assessment, information gained from the interviews were used to iteratively change the instrument. Additional questions assessed acceptability of screening within primary care and preferences around administration. Twenty-eight (28) caregivers were administered the questionnaire. Cognitive interviews conducted among caregivers and among 16 health providers and clinic staff resulted in the changes in wording and addition of examples in the items to increase face validity. In the final instrument, no new items were added; however, two items were merged and one item was split into three separate items. While there was a high level of acceptability of the overall questionnaire, some caregivers reported discomfort with the sexual abuse, separation from caregiver, and community violence items. Preference for methods of administration were split between tablet and paper formats. The final Pediatric ACE and other Determinants of Health Questionnaire is a 17-item instrument with high face validity and acceptability for use within primary care settings. Further evaluation on the reliability and construct validity of the instrument is being conducted prior to wide implementation in pediatric practice.

## Introduction

Adverse Childhood Experiences (ACEs) are stressful or traumatic events experienced before the age of 18 years and were found to have a dose-response relationship with numerous poor health outcomes in a landmark study conducted by the CDC and Kaiser [1]. Since the publication of the ACE Study in 1998, subsequent studies have demonstrated the broad prevalence of ACEs in the general population and have added to the evidence showing the graded association between ACEs and negative health and behavioral outcomes. Exposure to these adversities in childhood, without the buffering protection of a caregiver, may lead to changes in children’s developing brains resulting in a dysregulation of the stress response, impairment of executive functioning, changes to the endocrine and immune systems and to genetic regulatory mechanisms, increased risky behaviors, and difficulty with forming healthy relationships [2]. The exposure to ACEs is thought to have a cumulative effect over the life course and found to increase the likelihood of disease [3], starting as early as childhood. In children and adolescents, ACEs have been associated with fair or poor general health [4–5] illness requiring a doctor [5], infections [6–7] cognitive and developmental delays [8–10], fair or poor dental health [11], asthma [4], [12–14], Attention Deficit Hyperactive Disorder (ADHD) [4], [15], sleep disturbance [16–18], autism [4], overweight or obesity [19–21], violent behavior (delinquent behavior, bullying, physical fighting, dating violence, weapon-carrying) [22], suicide-related behaviors [23], and learning difficulties [4], [24].

Studies have shown that while the plasticity of the brain during early childhood and adolescence makes it particularly vulnerable to adversities, this is also an opportunity for intervention and treatment [25]. The temporality and intensity of ACEs occurrence during childhood is important and suggests that there is an opportunity to identify children at risk for accumulating ACEs and the associated negative health outcomes, to promote prevention and support the unfolding of resilience, and to develop targeted interventions for those identified as ‘at risk’.

The pediatric medical home is well positioned to embed universal screening for trauma and adversity in standard clinic flow. Because Pediatricians provide care for children and their families at regular intervals, patients and providers develop a trusting relationship, which facilitates screening and patient education about the impacts of adversity on health [26]. Current measures in practice are either limited to specific ACEs categories, such as child abuse, (e.g. the Childhood Trauma Questionnaire (CTQ)), or contain items that assess trauma more generally (e.g., The Traumatic Events Screening Inventory).

An age appropriate, tool that assesses cumulative adversity exposure is essential for identifying children at risk for developing poor health outcomes and tailoring appropriate interventions [27]. To address this need, researchers from the Bay Area Research Consortium on Toxic Stress and Health (BARC) designed and piloted a pediatric ACEs and other determinants of health screening instrument in a primary care pediatric setting that may ultimately be used to universally screen for childhood adversity. This pilot study had three aims:

1. To develop a core set of child adversity screening items from the existent literature and available screening instrument.
2. To assess the face validity of items through caregiver and provider interviews, including item clarity, content inclusion, and language equivalence (Spanish vs. English).
3. To document preferences around screening administration (modality, identification of specific items)

## Methods

### Design and development of the questionnaire

The BARC team formed a working group representative of the three consortium institutions: the Center for Youth Wellness (CYW), the University of California San Francisco (UCSF) and UCSF Benioff Children’s Hospital Oakland (BCHO), for the planning and implementation of the pilot study. The working group included physicians, nurse practitioners, mental health specialists/psychologists, content experts in ACEs research, and psychometricians. In 2015 the BARC working group conducted a review of existing ACEs and trauma screening tools used in current clinical practice. Concurrently, the BARC team interviewed six pediatricians and researchers in the fields of behavioral and mental health, policy, and education, who have modified and implemented versions of the ACEs questionnaire in their practice. In addition, the team searched websites of trauma screening tools including the National Child Traumatic Stress Network (NCTSN) and ACEs Connection. The team reviewed the tools’ content, and conducted a literature search to identify relevant validation studies. Authors were also contacted for additional details and updates on the identified tools. Thirty trauma screening tools were identified and reviewed. From this, the team excluded 25 tools. Reasons for tool exclusion included: focused on limited sources of trauma (n = 14); contained only general trauma questions (n = 6), use limited to a narrow or non-pediatric group (n = 15), and /or the tool was modified and reduced from the original ACEs questionnaire (n = 1). The remaining five relevant ACEs screening tools included: 1) ACE Study questionnaire [1], 2) the CYW ACE Questionnaires (CYW ACE-Q child and the CYW ACE-Q teen) [28], 3) the Jefferson County Public Health Prenatal Health History Questionnaire [29], and 4) the World Health Organization Adverse Childhood Experiences International Questionnaire (WHO ACE-IQ) [30].

The ACE Study questionnaire has ten categories representing three domains of ACEs: abuse, neglect, and family dysfunction. The ACE study found these ten categories to have strong dose-response associations with negative health outcomes. The Jefferson County Public Health Department ACE questionnaire is very similar to the ACE Study questionnaire with slight modifications in item presentation. The CYW ACE-Q (Child and Teen questionnaires) are a 17-item and a 19-item instrument respectively that contain all items in the ACE Study questionnaire along with the following supplemental categories hypothesized to be associated with a dysregulated stress response: serious medical procedures or life-threatening illness of the child, separation from caregiver via deportation or migration, discrimination, being a victim of bullying, and seeing or hearing neighborhood and school violence [31]. The WHO ACE-IQ is the most extensive tool with 36 items and covers all categories of the aforementioned screening tools, with the addition of items pertaining to marriage and exposure to war or collective violence.

Items from these selected instruments were organized by conceptual domains. Items within domains were reviewed on the basis of being 1) well formulated, 2) sensitive to low literacy audiences, and 3) conceptually distinct (item pertaining to one specific ACE item category) while maintaining high fidelity to ensure representation of the three conventional ACE domains (Abuse, Neglect, Household Dysfunction). Reviews were discussed within the BARC working group to reach full consensus across members. When deciding if there was a need to expand the definition of a category or add new categories, the working group applied the following criteria developed by experts from WHO [32] to evaluate each ACE item/category: biological relevance (i.e. evidence suggesting a biological stress response to exposure), low to moderate prevalence across communities, measurable, proximal in respect to causality, and policy relevance.

### Expansion of ACEs categories and supporting literature

Beginning with the three original ACE domains identified in the original ACE Study Questionnaire, which have been most rigorously studied and which have the strongest epidemiologic data linking ACE categories with health outcomes, a fourth domain of social determinants of health was considered. Four items were identified: food insecurity, housing instability, violence outside of the home, and experiences of discrimination. Consideration for addition of these items was informed by their inclusion in other ACE screeners (CYW ACE Q child and teen; WHO ACE-IQ) and reinforced by the growing literature supporting that they may act through the same dysregulated stress response mechanism, and are associated with a wide range of adverse endocrine, metabolic, mental and behavioral outcomes in animal models and in children and adolescents [15], [33–55]. To add to this literature a recent study investigated the inter-correlation among a 17-Item childhood Experiences Survey, including food insecurity and homelessness, among a low income population, and found that not only these items were prevalent, but they were also associated with the conventional 10 ACEs categories, in addition to be associated with the outcomes of interest [56]. Food insecurity and housing instability items were modified from the FIND (Family Information and Navigation Desk) screener for basic social needs [57]. The items of violence outside the home (defined as bullying, organized violent crime, police action, and acts of war or terrorism) and experiences of discrimination were modified from items of the WHO ACE-IQ and the CYW ACE-Q.

Prior to finalizing the questionnaire for the pilot study, the team made the following changes. First, the original ACE item of emotional abuse was expanded to encompass the complex nature of the item. Second, the original ACE item of incarceration was broadened to cover additional forms of separation from the caregiver and include examples such as foster care, immigration, and death of the caregiver. While the addition of these examples to the item was informed by the CYW ACE Q (child and teen), inclusion was also reinforced by the research showing their consistent linkages to health outcomes [58–66].

Third, the original ACE item of divorce was broadened to assess family cohesion (divorce being one example within this category). This expansion allowed for acknowledgement of the mixed literature linking divorce to health outcomes [67–70], while being sensitive to include the large numbers of families that are raising children while unmarried, and literature supporting the impact of changes in family cohesion beyond divorce on children’s development (e.g. change in partners) [71–74].

Finally, caregiver’s serious physical illness or disability was added under the domain of household dysfunction. While not represented in the reviewed ACE screeners, caregiver physical illness is included in many related trauma screening questionnaires used in clinical practice, including the Child and Adolescent Needs and Strengths—Trauma Comprehensive (CANS Trauma), the Traumatic Events Screening Inventory-Parent Report Revised (TESI-PRR), the Childhood Experience of Care and Abuse Questionnaire (CECA-Q), and the UCLA PTSD Reaction Index (Exposure portion). The decision for inclusion was also supported by findings from studies focused on the impact of caregiver physical illness and health outcomes in children [75–80], with a goal to assess its association with a dysregulation of the stress response.

### The pediatric ACE and other determinants of health questionnaire, first version

Utilizing the selected items and the findings from the literature review discussed to justify modifications, the group designed version 1 of the BARC Pediatric Adversity and Trauma Questionnaire (see Table 1 for the items, conceptual categories, and items’ sources). Version 1 of the questionnaire contained 16 items (scored yes/no), that assess the ten traditional ACEs categories, the items from the additional domain of social determinants of health (food insecurity, house instability, violence outside the house and discrimination), and an item on caregiver physical illness.

**Table 1:** Conceptual categories, initial items and sources.

Conceptual category	Item source	Item
Emotional abuse	CYW ACE-Q	Has a parent/caregiver ever sworn at, humiliated, put down or threatened to abandon your child?
Emotional neglect	CYW ACE-Q	Has your child ever felt unsupported, unloved and/or had no one to protect her/him?
Physical neglect	ACE Study	Has your child ever lacked appropriate care (for example, not being protected from unsafe situations, or not cared for when sick or injured even when you had the resources)?
Physical abuse	ACE Study/ CYW ACE-Q	Has any adult in the household ever pushed, grabbed, slapped or thrown something at your child? Or Has any adult in the household ever hit your child so hard that she or he had marks or was injured? Or Has any adult in the household ever threatened your child or acted in a way that made your child afraid that she or he might be hurt?
Sexual abuse	ACE Study/ CYW ACE-Q/ WHO ACE-IQ	Has anyone ever touched your child, in a way that was unwanted, or made your child feel uncomfortable? Or Has anyone ever asked your child to touch him or her in a way that was unwanted, or made your child feel uncomfortable? Or Has anyone ever attempted or actually had oral, anal, or vaginal sex with your child?
Separation from caregiver	CYW ACE-Q	Has your child ever been separated from his/her parent or caregiver due to prison, foster care, death, immigration, or any other reason?
Domestic violence	ACE Study	Has your child ever seen or heard a parent/caregiver being screamed at, sworn at, insulted or humiliated? Or Has your child ever seen or heard a parent/caregiver being slapped, kicked, punched beaten up or hurt with a weapon?
Caregiver’s substance use	CYW ACE-Q	Has your child ever lived with anyone who had a problem with drinking too much alcohol, used street drugs, or abused prescription medications?
Caregiver’s mental illness	CYW ACE-Q	Has your child ever lived with a parent/caregiver who was depressed, mentally ill or suicidal?
Caregiver’s physical illness	Study working group	Has your child ever lived with a parent/caregiver who had a serious medical illness or disability?
Community violence	CYW ACE-Q and WHO ACE-IQ	Has your child ever witnessed violence in your neighborhood, community or school? (for example, bullying, or organized violent crime, or police action, war or terrorism)?
		Has your child ever been a victim of violence in your neighborhood, community or school? (for example, bullying, or organized violent crime, or police action, war or terrorism)?
Discrimination	CYW ACE-Q and WHO ACE-IQ	Has your child experienced discrimination (for example being hassled or made feel inferior or excluded because of his/her race, ethnicity, gender identity, sexual orientation or religion)?
Housing instability	FIND*/Study working group	Has your child/family ever been homeless? OR Has your child ever had problems with housing (for example not having a stable place to live, faced eviction or foreclosure, or lived with multiple families)?
Food insecurity	FIND*/Study working group	Have you ever worried that your child did not have enough food to eat or that the food for your child would run out before you could buy more?
Low family cohesion	Study working group	Have there ever been significant changes in your family structure, such as a parent/caregiver got a divorce, or romantic partner moved in or out?

*FIND: Family Information and Navigation Desk’ needs assessment survey

The questionnaire was translated into Spanish by a certified translation company, and translated back to English by two native Spanish speaker study coordinators for equivalency and consistency. The questionnaire was then formatted into three mediums: paper, interview, and electronic (tablet) in order to test the instrument face validity (including clarity of content and comprehension), acceptability, and feasibility of its implementation in the UCSF Benioff Children’s Hospital Oakland pediatric primary clinic.

### Piloting of the questionnaire

#### Study participants

A convenience sample was drawn from families receiving care at the UCSF Benioff Children’s Hospital Oakland, Claremont Primary Care Clinic. Caregivers were called on the phone or approached during their well child check, then screened for eligibility. Eligibility criteria included: caregiver aged 18 years or older, being the primary caregiver of a child age 12 or under, speaking English and/or Spanish, and having no other child enrolled in the study. Eligible caregivers screened in the clinic who agreed to participate in the study were enrolled, and eligible caregivers screened over the phone were scheduled to come to the clinic for enrollment. In addition to caregivers, the study included a convenience sample of the UCSF BCHO Primary Care clinic providers and staff across clinic roles to assure breadth of response.

#### Procedures and measures

All eligible and interested participants met with a study coordinator at the clinic. After having the study explained and providing written consent, enrolled caregivers were randomly assigned to complete the Pediatric ACE and other Determinants of Health Questionnaire on one of the three modalities in their preferred language (English or Spanish): paper, interview (questions verbally read out loud to participants by research coordinator), and electronic (tablet). The electronic format had an audio feature—meaning that participants could play the audio recordings of the questions. A cognitive interview directly followed the questionnaire. Interviews focused on caregivers: (1) comfort answering the questions within the healthcare setting, (2) item clarity and difficulty responding to items, (3) item understanding and intended meaning (including what participants felt the question was asking and the experiences that answering “yes” would include or not include), and (4) whether there were any life experiences they felt were missing or not assessed. In instances in which confusion or lack of clarity emerged, the interviewer prompted for targeted feedback for improving the survey. Bilingual participants were additionally shown both the English and Spanish versions of the survey and provided feedback on language equivalence vs. discordance. Caregivers were then presented with all three modalities of the questionnaire (interview, tablet, and paper) and asked their preferred modality.

Enrolled clinic providers and staff were presented with the three modalities of the questionnaire, asked their preferred modality for use with their patients, and inquired feedback around presentation, clarity of item wording, conceptual categories not covered or represented, and comfort with administration within their healthcare setting.

The interviews were audio recorded, and the investigators took additional observation notes.

#### Analysis

Broad thematic analysis of the interview notes and the tape recordings was conducted to distill caregivers and providers’ thoughts and attitudes toward the ACEs screening. Review of audio recordings and interview notes were discussed extensively at weekly working group meetings, and decisions on implementing changes in the questionnaire were made with full group consensus. Caregivers and providers and staff’s feedback on how to clarify or improve items received throughout the week was discussed and incorporated in the questionnaire during the working group meeting at the end of each week in rapid and iterative cycles. Changes made to the questionnaire were then tested on subsequent enrolled caregivers. Seven weekly rapid cycles were carried out until saturation of themes and high clarity was reached.

#### Ethical considerations

The study protocol was reviewed and approved by the Children’s Hospital & Research Center Oakland’s institutional review board. All participants signed a consent form, and were provided a copy. Caregivers enrolled received a $25 gift card incentive. Data were de-identified and labeled with study IDs.

## Results

**Fig 1:**
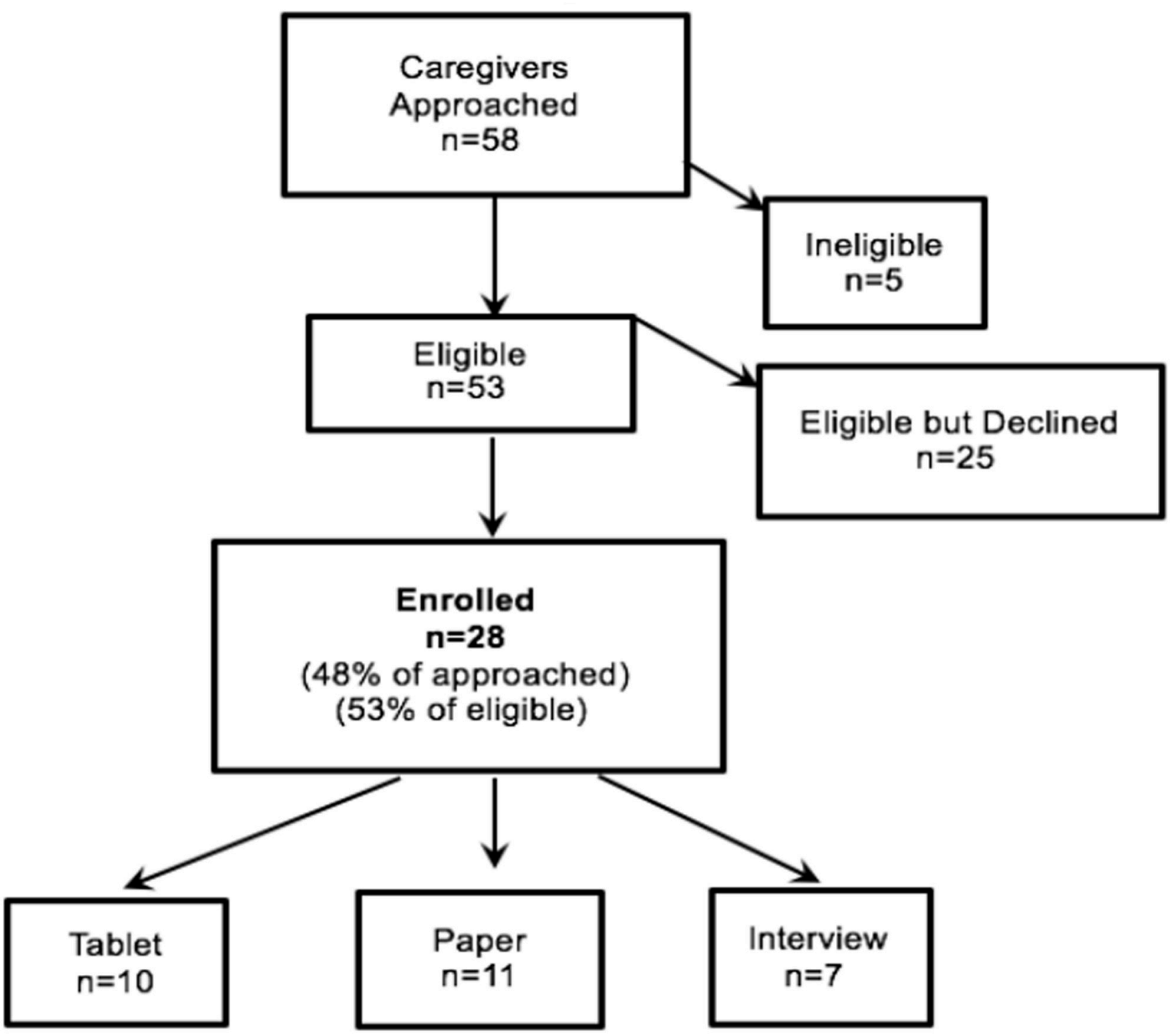
Consort diagram of study recruitment (caregivers).

**Fig 2:**
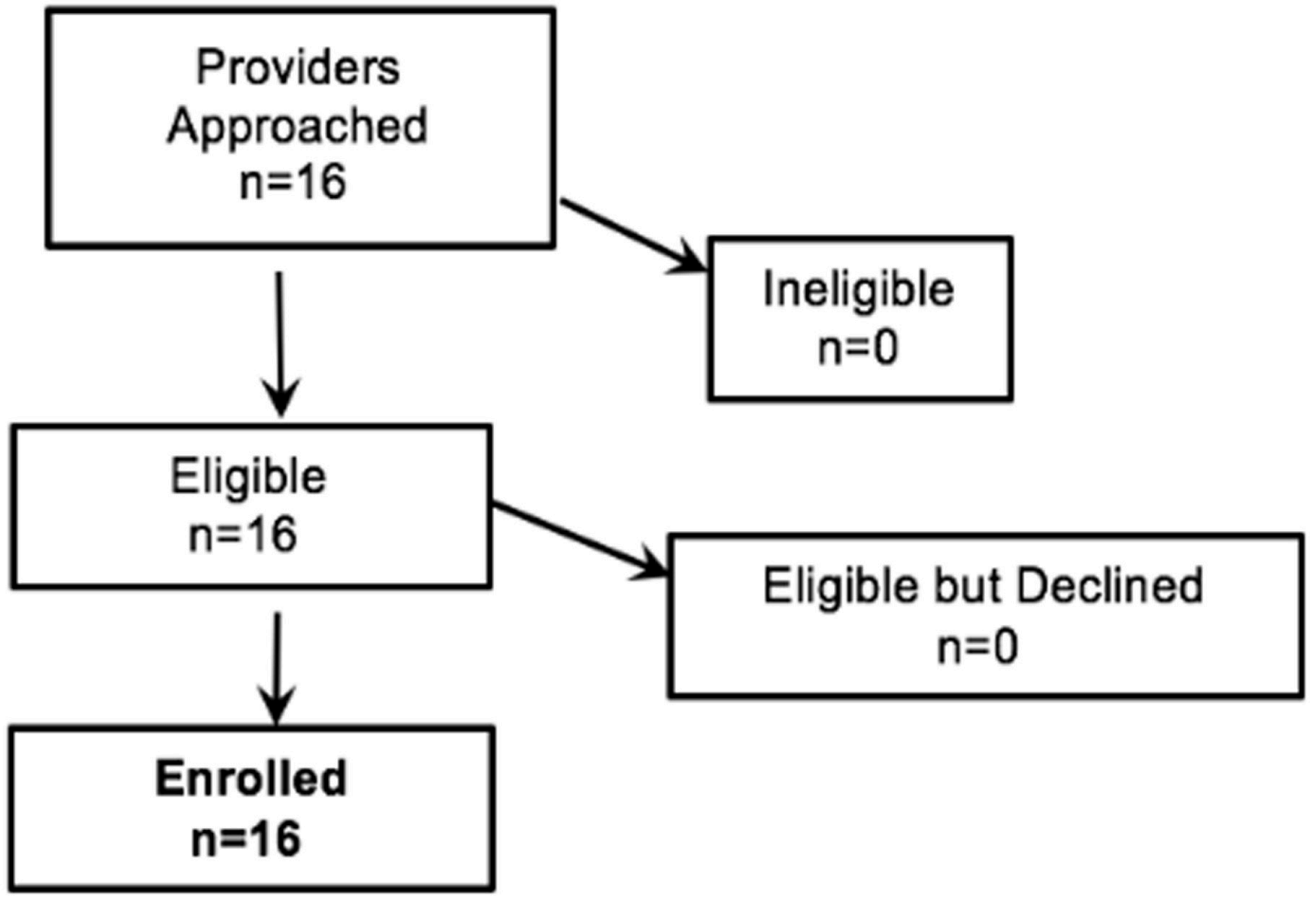
Consort diagram of study recruitment (providers and staff).

Between July 1 and August 26, 2016, 28 caregivers and 16 health providers and clinic staff participated (Figs 1 and 2). The majority of caregivers were the child’s biological parent (89.3%); female (92.9%), and identified as either White Hispanic (42.9%) or Black non-Hispanic (39.3%). Of the 28 caregivers, 57.1% attended some college or graduated from college and 46.4% had an annual income of less than $25,000. English was the language spoken by 57.1%, followed by Spanish (35.7%), and 7.1% were bilingual. Children had a mean age of 6 years (SD: 3.1), 50% female. Thirty nine percent were identified by caregivers as White Hispanic, 32.1% as Black non-Hispanic, and 14.3% as mixed ethnicity. (See Table 2).

**Table 2:** Study participants’ characteristics.

Participants	Caregivers (n = 28)	Children (n = 28)	Providers and staff (n = 16)
Gender			
Female	92.9% (n = 26)	50% (n = 14)	75% (n = 12)
Male	7.1% (n = 2)	50% (n = 14)	25% (n = 4)
Age (mean)	39.2% (SD = 8.7)	6 years (SD: 3.1)	
Relationship to child			
Parent	89.3 (n = 25)		
Grandparent	3.5% (n = 1)		
Legal guardian	7.1% (n = 2)		
Ethnicity			
Black non-Hispanic	39.3% (n = 11)	32.1%(n = 9)	31.3% (n = 5)
White non-Hispanic	3.5% (n = 1)	21.4% (n = 6)	37.5% (n = 6)
White Hispanic	42.9% (n = 12)	7.1% (n = 2)	18.8% (n = 3)
Black Hispanic	7.1% (n = 2)	39.3% (n = 11)	
Mixed	3.5% (n = 1)	32.1%(n = 9)	
Marital status			
Married/long-term couple	50% (n = 14)		
Single/never married	28.6% (n = 8)		
Divorced/separated	10.7% (n = 3)		
Education			MD: 44% (n = 7)
College graduate or higher	17.9% (n = 5)		LVN: 18.8% (n = 3)
Some College	39.3% (n = 11)		NP: 6.3% (n = 1)
High school or GED	32.1% (n = 9)		SW: 6.3% (n = 1)
Middle school or lower	10.7% (n = 3)		AS: 12.5% (n = 2)
Income			
$1000–15000	17.9% (n = 7)		
$15001–25000	21.4% (n = 6)		
$25001–35000	14.3% (n = 4)		
$35001–45000	21.4% (n = 6)		

MD: Medical Doctor; LVN: Licensed Vocational Nurse; NP: Nurse Practitioner: SW: Social Worker; AS: Administrative Support

The 16 health providers and clinic staff members enrolled included: 44% Medical Doctors (MD), 18.6% Licensed Vocational Nurse (LVN), 6.3 Nurse Practitioners (NP), 6.3% Social Workers, 12.4% Administrative Support. Females were the most represented (75%); 37.5% were White non-Hispanic, 31.3% Black Hispanic, 18.8% White Hispanic. The majority (56.3%) had been working for 10 years or more at the clinic.

### Rapid cycle testing of the questionnaire

#### Face Validity and item Clarity/Understanding

Overall, participants reported very few areas of confusion in which they were unclear how to answer or could not answer an item. However, interview probes around item understanding uncovered several areas in which the wording did not adequately encompass the majority of experiences as intended by the authors, or the converse, included a wider range of experiences than intended or supported by the literature. To address these issues and achieve a high level of face validity and item understanding, changes to item wording were made in an iterative fashion (Version 1 through Version 8. Changes included replacing pronouns such as his or her by “their” or “your child” to clarify items and to be inclusive of all genders. Specific words were removed or replaced per participants’ suggestions to heighten participants’ understanding and encompass their experiences. For example, in the caregiver’s mental illness item, “depressed, mentally ill” was replaced with “mental health issues” with specific examples added. In the caregiver’s incarceration item, “jail” was added. In other items, temporality was added to capture both current and past experiences of their child. No additional categories of trauma or adversity were consistently identified as missing by caregivers or providers and staff. Thus, no new items were added during the pilot.

Further changes improved clarity by combining or separating item content. The community violence included an item on witnessing a violence, and an item on being a victim of violence. In response to multiple participants experiencing difficulty in cognitively separating these two experiences, these items were combined to improve understanding. The separation item initially included reasons due to prison, foster care immigration, and death. The working group decided to disaggregate this item to have separation from caregiver due to caregiver’s death as standalone item to acknowledge the amplitude and potentially unique impact on the child [64–66] and for tailoring of intervention. Similarly, the item of separation due to prison was made standalone given the growing literature demonstrating independent effect [81–83] and to allow for intervention tailoring. The separation due to foster care and immigration became one item to allow its testing to confirm or refute their association with health outcomes found in other studies.

English/Spanish equivalency was obtained by presenting both the English and Spanish questionnaires to bilingual participants. Items were compared side by side to attest the meanings remained consistent across languages. This allowed for specific changes in words or phrases to achieve equivalence in meaning. The comparison by bilingual participants also allowed for improving clarity in other expressions that are influenced by culture. For instance, “romantic relationship” in the Spanish questionnaire was highlighted to be two words with the same meaning. The phrase was changed to permit the English equivalency.

#### Acceptability of screening administration

Overall, all three modalities of administration (paper, interview, tablet) were seen as acceptable by the majority of participants. Where a clear preference was stated, preference was mixed across paper, interview and tablet. Twenty percent of caregivers stated a clear preference for tablet with similar percentage stating a sole preference for interview or paper. Only one participant used the audio feature. Improvements to tablet screen interface during rapid cycling improved caregivers’ reactions to tablet. The improvements included an addition of a progress bar to show how far the respondent has gone through the questions, a change in layout, shading, font and the “next button” size, and a decrease in the number of questions per screen page. Providers and staff abstained from stating a preference in modality, leaving the choice to caregivers and their preference.

#### Overall comfort with the questionnaire

Both caregivers and providers were asked about their comfort in responding to and administering the questionnaire in the primary care setting during the interview following the survey. About half of the caregivers, particularly those endorsing questionnaire items on abuse, separation from caregiver, and community violence, expressed some discomfort. The items generated tearful, emotional responses in some caregivers. They reported that the items triggered memories of their own personal painful experiences as a child. Nevertheless, no caregiver wished to discontinue; all completed the questionnaire. They expressed gratitude that someone was asking questions about adversity, and displayed an understanding of the necessity to share their current family experiences. They highlighted the relevance of informed health providers to receiving targeted support in these areas. The quality of the relationship with their provider was cited numerous times as a supporting reason to engage more deeply. Caregivers felt comfortable at discussing their experiences with providers as long as there was a trusted relationship. Providers uniformly expressed comfort with administering the questionnaire and discussing the results with caregivers. Concerns with the questionnaire included time constraints and availability of resources to address positive results. All participants agreed on the reality of the issues inquired by the questionnaire and the need to address them in order to achieve an overall health.

#### The pediatric ACE and other determinants of health questionnaire, final version

See S1 Appendix for the final version of the pediatric ACE and other determinants of health Questionnaire.

## Discussion

To date, there is no known existing validated questionnaire designed to comprehensively screen young children and adolescents for exposure to ACEs in a pediatric primary care setting. Drawing from the adult ACE and other adversity and trauma screening tools, a literature review, the WHO criteria and the team’s experience, we developed the first pediatric ACE and other determinants of health questionnaire. Caregivers and primary care providers and staff’s insights through cognitive interviews allowed the team to test the questionnaire for item clarity, content inclusion, and English and Spanish language equivalency. The team’s process of forming a working group and utilizing empirical evidence in developing the questionnaire is common. This approach was informed by the development of the WHO International ACEs questionnaire [32], the Obsessive Belief Questionnaire [84], and the Alcohol, Smoking and Substance Involvement Screening Test (ASSIST)[73].

Patients and stakeholders’ inputs to the development of screening tool have proven significant in various studies and specialties [85–87]. The intended users for the questionnaire being caregivers and primary care providers, it was imperative to have their insights. The administration of the questionnaire to the caregivers gave users the opportunity to experience the process and provide invaluable insights that directly improved item clarity and comprehension for the target audience that could not have been obtained otherwise. The decision to administer the questionnaire to caregivers and parents only was influenced by the study design and the study sample. The study sample included children aged 3 months to 11.99 years and their caregivers. Administrating the questionnaire to both children and caregivers wouldn’t have been appropriate for the children whose mean age was 6 years. The reading level of the questionnaire was set to grade 6.

While preference for administration was spread across modalities, all participants found the screening acceptable. Providers and staff expressed openness and the benefits of screening and identifying ACEs in primary care practices; caregivers were receptive to the questionnaire, and felt comfortable at discussing ACEs with their pediatricians. Caregivers underlined the importance of the relationship and trust-building with the providers. Similar results were found in other studies [87–89].

These findings reinforce the AAP’s statement that pediatric care settings are ideal for adversity screening because of the trusting relationship caregivers have with their pediatricians [26]. While both caregivers and providers were agreeable to adversity screening in pediatric primary care setting, providers felt they needed additional resources to be able to meet families’ needs. All participants agreed that something needed to be done after screening. In places where ACEs screening is standard practice, processes are put in place to make sure that families that screen positive receive appropriate care with the support of an integrated multidisciplinary team that works hand in hand to make sure families are cared for from screening to intervention [90].

## Conclusion

The collaborative approach supported by empirical evidence and a rapid cycle testing through cognitive interviews proved to be effective in developing a pediatric adversity questionnaire. Using this approach, we developed the BARC Pediatric ACE and other Determinants of Health Questionnaire and conducted its face validity. The 17-item instrument is being validated for content and construct in a longitudinal study. Once validated, the tool will allow the screening for exposure to childhood adversity for early detection of exposure and prevention of future occurrences. This pilot study has also shown that screening for childhood adversity is acceptable and feasible in primary care setting with appropriate resources.

## Supporting information

S1 Appendix Final pediatric ACE and other determinants of health questionnaire. (DOCX)

S1 File FIT caregiver consent. (DOCX)

S2 File FIT provider consent. (DOCX)
