# Supplementary material for: Development and implementation of a pediatric adverse childhood experiences (ACEs) and other determinants of health questionnaire in the pediatric medical home: A pilot study
Source: PLoS One. 2018 Dec 12;13(12):e0208088. doi: 10.1371/journal.pone.0208088 (PMC6291095; doi:10.1371/journal.pone.0208088)
Supplement: S1 Appendix — (DOCX) [file pone.0208088.s001.docx]

**S1 Appendix: Final Pediatric ACE and other determinants of health questionnaire**

| **BARC Pediatric ACE and other Determinants of Health Questionnaire** | |
| --- | --- |
| **Study ID #:_______________________Today’s date__________________**  **To be completed by Caregiver** | |
| **Many families experience stressful life events. Over time these experiences can affect your child's health and wellbeing. We would like to ask you questions about your child so we can help them be as healthy as possible. At any point in time since your child was born, has your child seen or been present when the following experiences happened? Please include past and present experiences.**  **Please note, some questions have more than one part separated by “OR.” If any part of the question is answered “Yes, “then the answer to the entire question is “Yes.”** | |
| 1. Has your child ever lived with a parent/caregiver who went to jail/prison? | Yes No  ☐ ☐ |
| 1. Do you think your child ever felt unsupported, unloved and/or unprotected? | Yes No  ☐ ☐ |
| 1. Has your child ever lived with a parent/caregiver who had mental health issues? (for example, depression, schizophrenia, bipolar disorder, PTSD, or an anxiety disorder) | Yes No  ☐ ☐ |
| 1. Has a parent/caregiver ever insulted, humiliated, or put down your child? | Yes No  ☐ ☐ |
| 1. Has the child’s biological parent or any caregiver ever had, or currently has a problem with too much alcohol, street drugs or prescription medications use? | Yes No  ☐ ☐ |
| 1. Has your child ever lacked appropriate care by any caregiver (for example, not being protected from unsafe situations, or not cared for when sick or injured even when the resources were available)? | Yes No  ☐ ☐ |
| 1. Has your child ever seen or heard a parent/caregiver being screamed at, sworn at, insulted or humiliated by another adult?   Or  Has your child ever seen or heard a parent/caregiver being slapped, kicked, punched beaten up or hurt with a weapon? | Yes No  ☐ ☐ |
| 1. Has any adult in the household often or very often pushed, grabbed, slapped or thrown something at your child?   Or  Has any adult in the household ever hit your child so hard that your child had marks or was injured?  Or  Has any adult in the household ever threatened your child or acted in a way that made your child afraid that they might be hurt? | Yes No  ☐ ☐ |
| 1. Has your child ever experienced sexual abuse? For example, anyone touched your child or asked your child to touch that person in a way that was unwanted, or made your child feel uncomfortable, or anyone ever attempted or actually had oral, anal, or vaginal sex with your child? | Yes No  ☐ ☐ |
| 1. Have there ever been significant changes in the relationship status of the child’s caregiver(s)? For example, a parent/caregiver got a divorce or separated, or a romantic partner moved in or out? | Yes No  ☐ ☐ |
| **Write the total number of Yeses in this first section here:** |  |

| 1. Has your child ever seen, heard, or been a victim of violence in your neighborhood, community or school? (for example, targeted bullying, assault or other violent actions, war or terrorism) | Yes No  ☐ ☐ |
| --- | --- |
| 1. Has your child experienced discrimination (for example being hassled or made to feel inferior or excluded because of their race, ethnicity, gender identity, sexual orientation, religion, learning differences, or disabilities)? | Yes No  ☐ ☐ |
| 1. Has your child ever had problems with housing (for example being homeless, not having a stable place to live, moved more than two times in a six-month period, faced eviction or foreclosure, or had to live with multiple families or family members)? | Yes No  ☐ ☐ |
| 1. Have you ever worried that your child did not have enough food to eat or that the food for your child would run out before you could buy more? | Yes No  ☐ ☐ |
| 1. Has your child ever been separated from their parent or caregiver due to foster care, or immigration? | Yes No  ☐ ☐ |
| 1. Has your child ever lived with a parent/caregiver who had a serious physical illness or disability? | Yes No  ☐ ☐ |
| 1. Has your child ever lived with a parent or caregiver who died? | Yes No  ☐ ☐ |
| **Write the total number of Yeses in this second section here:** |  |
