# Supplementary material for: Development and implementation of a pediatric adverse childhood experiences (ACEs) and other determinants of health questionnaire in the pediatric medical home: A pilot study
Source: PLoS One. 2018 Dec 12;13(12):e0208088. doi: 10.1371/journal.pone.0208088 (PMC6291095; doi:10.1371/journal.pone.0208088)
Supplement: S1 File — (DOCX) [file pone.0208088.s002.docx]

**
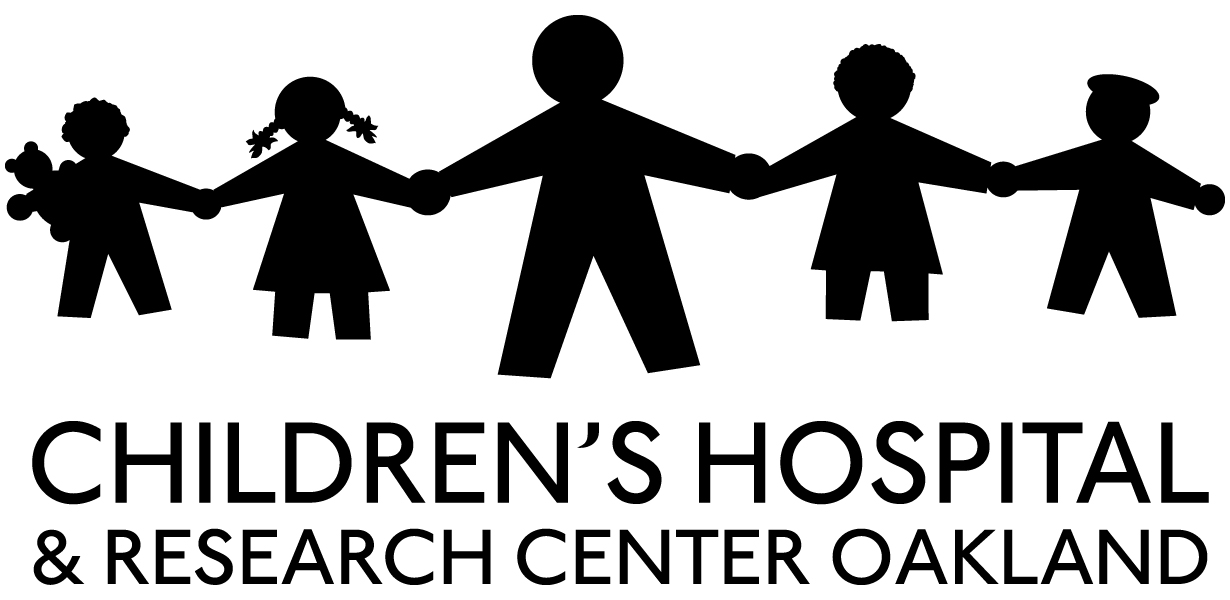
**

**CONSENT TO PARTICIPATE IN A RESEARCH STUDY – Parent/Caregiver**

**NAME OF THE STUDY: Bay Area Research Consortium on Toxic Stress and Health** *FIT: Feasibility of Implementing* an ACEs (Adverse Childhood Experiences) *Screening Tool*

**WHAT IS THIS STUDY ABOUT?**

This study aims to get parent/caregiver input to help us make a screening questionnaire to use in clinic. The questionnaire is about Adverse Events (ACEs) which are hard events that may have happened to children like experiencing violence or abuse and other hard experiences. Many families experience stressful life events. Overtime, these experiences may affect a child’s health and wellbeing. This study will help us make a useful questionnaire so that in the future we can identify and support children who experience ACEs.

**WHO IS PAYING FOR THIS STUDY?**

This study is paid for by Tara Health Foundation.

**HOW MANY PEOPLE WILL BE IN THIS STUDY?**

About 32 parent/caregivers will be in this study at UCSF Benioff Children’s Hospital Oakland Claremont Clinic.

**WHAT WILL HAPPEN IN THE STUDY?**

If you agree to be in this study, the following will happen:

You will be asked to complete a survey about your and your child’s experiences. Then you will be asked your opinion about the survey. We will also ask about your feelings as you were taking the survey. The interview may take up to 1 hour.

**WHAT ARE THE RISKS OR POSSIBLE SIDE EFFECTS OF THIS STUDY?**

Answering the survey questions may make you feel uncomfortable or raise unpleasant memories. You are free to skip any questions.

**ARE THERE BENEFITS TO BEING IN THE STUDY?**

There will be no direct benefit to you from participating in the study. However, your input will help us create programs for children who have experienced adverse events.

**WHAT OTHER CHOICES DO I HAVE?**

You are under no obligation to participate in this study. If you decide not to take part in this study there will be no penalty to you or your child. Your child may continue to receive care at UCSF Benioff Children’s Hospital Oakland Claremont Clinic.

**HOW WILL MY PRIVACY BE PROTECTED?**Study records that identify you will be kept confidential as required by law. Federal Privacy Regulations protect your privacy, restrict who is allowed to look at your records, and require security to protect your records. Except when required by law, you will not be identified by name, social security number, address, telephone number, or any other direct personal identifier in study records shared outside of UCSF Benioff Children’s Hospital Oakland. For research records shared outside of Children’s Hospital & Research Center Oakland, you will be given a code number. The list that can match you to the code number will be kept in a locked file in the Principal Investigator's office.

**DO I HAVE TO PAY TO BE IN THE STUDY?** There will be no charge to participate in this study.

**WILL I BE PAID FOR BEING IN THE STUDY?** You will be given a $50 gift card from Target as a thank-you for your time and participation.

**WHAT IF I HAVE QUESTIONS OR PROBLEMS?**

If you have any questions about the research, either before deciding whether to participate or during this study, please call Dr. Long at 510-428-3129. If you wish to speak to someone not associated with this study about complaints or your rights as a research participant, you may contact the Institutional Review Board (that reviews the research to protect your rights) at:

UCSF Benioff Children's Hospital Oakland

IRB Office

747 52^nd^ Street

Oakland, CA 94609

(510) 428-3754

**WHAT ARE MY RIGHTS? DO I HAVE TO AGREE TO THIS STUDY?**

You do not have to be in this research study. It is your choice. If you agree to be in this study and later change your mind, you may stop at any time. No matter what your decision is, including if you withdraw, there will be no penalty. If you wish to be in the study, please sign this form.

**CONSENT TO BE A RESEARCH PARTICIPANT:**

Your signature below indicates that you agree to be in this study. You will be given a signed copy of this form to keep.

Date:____________________ Name (Print) _____________________________

Signature: ___________________________

**STATEMENT OF INVESTIGATOR (or person obtaining consent):**

I have carefully explained to the participant all of the information in the consent form.

Date:____________________ Name (Print) _____________________________

Signature: ___________________________
