# Supplementary material for: Development and implementation of a pediatric adverse childhood experiences (ACEs) and other determinants of health questionnaire in the pediatric medical home: A pilot study
Source: PLoS One. 2018 Dec 12;13(12):e0208088. doi: 10.1371/journal.pone.0208088 (PMC6291095; doi:10.1371/journal.pone.0208088)
Supplement: S2 File — (DOCX) [file pone.0208088.s003.docx]

**
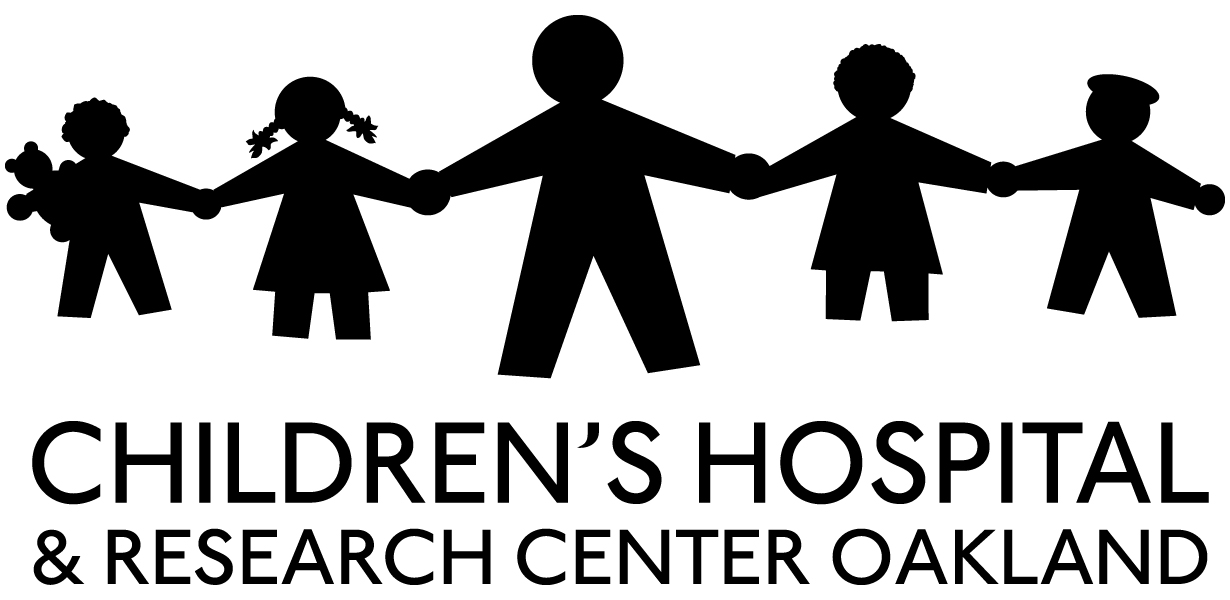
**

**CONSENT TO PARTICIPATE IN A RESEARCH STUDY – Provider/Staff**

**NAME OF THE STUDY: Bay Area Research Consortium on Toxic Stress and Health** *FIT: Feasibility of Implementing* an ACEs (Adverse Childhood Experiences) *Screening Tool*

**WHAT IS THIS STUDY ABOUT?** This study aims to understand ways to support clinicians and staff to identify, understand, and address Adverse Childhood Experiences in a Pediatric Primary Care setting.

**WHO IS PAYING FOR THIS STUDY?**

This study is paid for by Tara Health Foundation.

**HOW MANY PEOPLE WILL BE IN THIS STUDY?**

About 20 clinicians and support staff will be in this study at UCSF Benioff Children’s Hospital Oakland Primary Care Clinic.

**WHAT WILL HAPPEN IN THE STUDY?**

If you agree to be in this study, the following will happen: You will be asked to review/critique potential ACES screening questions and three possible modalities for screening your patients for Adverse Childhood Experiences. You will then be asked a series of questions aimed at eliciting your knowledge, capacity and comfort in talking with families about ACES. The interview may take up to 1 hour.

**WHAT ARE THE RISKS OR POSSIBLE SIDE EFFECTS OF THIS STUDY?**

Reviewing the ACES questions may make you feel uncomfortable or raise unpleasant feelings related to your own past experiences or experiences you have had with patients.

**ARE THERE BENEFITS TO BEING IN THE STUDY?**

Your input will assist in the development of programs to serve patients who have experienced Adverse Childhood Experiences.

**WHAT OTHER CHOICES DO I HAVE?**

You are under no obligation to participate in this study.

**HOW WILL MY PRIVACY BE PROTECTED?** Study records that identify you will be kept confidential as required by law. Federal Privacy Regulations protect your privacy, restrict who is allowed to look at your records, and require security to protect your records. Except when required by law, you will not be identified by name, social security number, address, telephone number, or any other direct personal identifier in study records shared outside of UCSF Benioff Children’s Hospital Oakland.

747 Fifty Second Street ● Oakland, CA 94609-1809

**510-428-3000**

www.childrenshospitaloakland.org

**DO I HAVE TO PAY TO BE IN THE STUDY?** There will be no charge to participate in this study.

**WILL I BE PAID FOR BEING IN THE STUDY?** You will be given a $20 gift card from PEETS as a thank you for your time and participation.

**WHAT IF I HAVE QUESTIONS OR PROBLEMS?**

If you have any questions about the research, either before deciding whether to participate or during this study, please call Dr. Long at 510-428-3129. If you wish to speak to someone not associated with this study about complaints or your rights as a research participant, you may contact the Institutional Review Board (that reviews the research to protect your rights) at:

UCSF Benioff Children's Hospital Oakland

IRB Office

747 52^nd^ Street

Oakland, CA 94609

(510) 428-3754

**WHAT ARE MY RIGHTS? DO I HAVE TO AGREE TO THIS STUDY?**

You do not have to be in this research study. It is your choice. If you agree to be in this study and later change your mind, you may stop at any time. No matter what your decision is, including if you withdraw, there will be no penalty. If you wish to be in the study, please sign this form.

**CONSENT TO BE A RESEARCH PARTICIPANT:**

Your signature below indicates that you agree to be in this study. You will be given a signed copy of this form to keep.

**Subject**:

| Date: |  | Time: |  | Signature: |  |
| --- | --- | --- | --- | --- | --- |
|  |  |  | (hh:mm) |  |  |
|  |  |  |  | Name (print): |  |

**STATEMENT OF INVESTIGATOR (or person obtaining consent):**

I have carefully explained to the participant all of the information in the consent form.

| Date: |  | Time: |  | Signature: |  |
| --- | --- | --- | --- | --- | --- |
|  |  |  | (hh:mm) |  |  |
|  |  |  |  | Name (print): |  |
